# An automatic method for robust and fast cell detection in bright field images from high-throughput microscopy

**DOI:** 10.1186/1471-2105-14-297

**Published:** 2013-10-04

**Authors:** Felix Buggenthin, Carsten Marr, Michael Schwarzfischer, Philipp S Hoppe, Oliver Hilsenbeck, Timm Schroeder, Fabian J Theis

**Affiliations:** 1 Institute of Computational Biology, Helmholtz Center Munich, 85764 Neuherberg, Germany; 2 Stem Cell Dynamics Research Unit, Helmholtz Center Munich, 85764 Neuherberg, Germany; 3 Department of Mathematics, Technical University Munich, 85748 Garching, Germany; 4 Department of Biosystems Science and Engineering (D-BSSE), ETH Zurich, 4058 Basel, Switzerland

## Abstract

**Background:**

In recent years, high-throughput microscopy has emerged as a powerful tool to analyze cellular dynamics in an unprecedentedly high resolved manner. The amount of data that is generated, for example in long-term time-lapse microscopy experiments, requires automated methods for processing and analysis. Available software frameworks are well suited for high-throughput processing of fluorescence images, but they often do not perform well on bright field image data that varies considerably between laboratories, setups, and even single experiments.

**Results:**

In this contribution, we present a fully automated image processing pipeline that is able to robustly segment and analyze cells with ellipsoid morphology from bright field microscopy in a high-throughput, yet time efficient manner. The pipeline comprises two steps: (i) Image acquisition is adjusted to obtain optimal bright field image quality for automatic processing. (ii) A concatenation of fast performing image processing algorithms robustly identifies single cells in each image. We applied the method to a time-lapse movie consisting of ∼315,000 images of differentiating hematopoietic stem cells over 6 days. We evaluated the accuracy of our method by comparing the number of identified cells with manual counts. Our method is able to segment images with varying cell density and different cell types without parameter adjustment and clearly outperforms a standard approach. By computing population doubling times, we were able to identify three growth phases in the stem cell population throughout the whole movie, and validated our result with cell cycle times from single cell tracking.

**Conclusions:**

Our method allows fully automated processing and analysis of high-throughput bright field microscopy data. The robustness of cell detection and fast computation time will support the analysis of high-content screening experiments, on-line analysis of time-lapse experiments as well as development of methods to automatically track single-cell genealogies.

## Background

### Advances in high-throughput microscopy

In the last decade, improvements in automated microscopy have enabled researchers to conduct two new types of experiments. On the one hand, high-content screening approaches allow to automatically quantify phenotypic changes of cells under a large range of different environmental conditions [1]. This technique is extensively used in pharmaceutics, for example in drug development and evaluation [2]. On the other hand, high-throughput time-lapse microscopy is a powerful tool to follow hundreds of single cells over many days and has been successfully applied in the field of hematopoietic research [3-5]. Equipped with appropriate cell tracking and image processing capabilities, this approach allows to analyze single cell dynamics in a quantitative and time-resolved manner [6,7].

A bottleneck in the analysis of high-throughput microscopy is the availability of suitable automatic processing tools that make the huge amount of information that is hidden in the data accessible [8]. Most experimental setups are highly specialized for the study of a single process of interest. Thus, different combinations of objectives, cameras or cell culture plates are used for different experiments, leading to great variability of images even for similar experimental setups. In addition, the large amount of images that is taken in high-throughput microscopy, either of many different cell culture plates or over a long time range adds to this variability. Since no standardized methods for the acquisition of long-term single cell microscopy exist, bioimage informatics methods have to be adapted for each setup. However, even in a single long-term experiment, images can vary to a degree that makes a unique parameterization for the whole movie challenging. In order to receive robust results from automated image processing it is advantageous to develop the methodology in close collaboration with experimentalists, resulting in an approach that performs best on the given data set.

### Fluorescence-based high-throughput image processing

Several computational methods for automatic processing of high-throughput microscopy experiments have been proposed. For example, Fenistein and colleagues [9] developed an automatic method for the segmentation of cell nuclei in fluorescence images for different cell lines in dilution experiments and report an average cell recognition rate of 95%. Knapp et al. [10] employed a method to identify single cells in two-channel RNAi screens and used this information to improve the statistical power of the analysis. Both applications demonstrated the feasibility of automatic high-throughput image processing methods on large amounts of fluorescent images.

The framework *CellProfiler*[11] is a great example of how automated image analysis can be made accessible for a broad range of users, not only specialists. The intuitive GUI and wealth of different implemented methods has led to frequent usage (601 citations, as of January 2013), where most applications relate to the analysis of fluorescence images.

### Analysis of bright field images in the context of high-throughput experiments

In general, analyzing cells in the bright field channel holds several benefits: (i) Since acquisition of fluorescent images over longer time spans (days) and with short intervals (minutes) is difficult due to photo toxicity, in long-term time-lapse experiments it is useful to employ bright field images to track cell genealogies [5]. (ii) Quantifying cell morphology in bright field images yields the possibility to measure more descriptive features such as texture and shape simultaneously. In addition, the fluorescence staining of e.g. the cytoplasm could be incomplete or less reliable on a special cell type, which would introduce bias in the later analysis. (iii) Due to the limited number of different fluorescent dyes that can be detected simultaneously, it is desirable to use as many channels as possible for fluorescence detection. Instead of losing a channel for nuclear staining to identify cells, one could observe expression of several fluorescent proteins or lineage markers and detect cell outlines in the bright field channel [3].

A few publications have already shown that incorporating data from bright field microscopy can yield interesting results. By quantifying the morphological behavior of tracked neural progenitors over time, Cohen et al. [12] could predict the most likely lineage decision of those cells. Scherf et al. [13] recently published a method to quantify changes in morphology of colonies of embryonic stem cells under different environmental conditions. In the field of high-content screening, Waehlby et al. [14] developed a toolbox based on *CellProfiler* to automatically quantify effects of different treatments to *C. elegans* populations. Adiga and colleagues [15] classified the infection state of macrophages by segmentation and morphological quantification in amplitude contrast bright field images.

### Challenges in high-throughput processing of bright field images

However, the development of an automated processing method for high-throughput bright field experiments is more demanding than in the fluorescence case and holds several challenges. Cells imaged by bright field microscopy exhibit heterogeneous intensity levels and are often badly contrasted. In addition, differences in illumination over time and across the cell culture plate hamper the ability to specify a global set of parameters for cell detection algorithms over the whole experiment. This prevents the application of available automatic image processing frameworks, which are mostly developed to perform well on fluorescent images. Despite the large amount of methods that are implemented in frameworks like *CellProfiler*, the available algorithms for illumination correction and segmentation do not perform well enough to achieve satisfying results on many high-throughput bright field microscopy experiments.

By employing active contour and level set methods, many issues of cell segmentation in bright field or phase contrast images have already been solved [16,17]. For example, Ambuehl et al. [18] demonstrated the very accurate tracking of a single cell in phase-contrast microscopy images. Ali et al. [19] developed a method that combined out of focus image acquisition and segmentation by level sets to identify outlines of adherent cells. However, these approaches are computationally expensive and often highly parameter-depended, which prevents the application in high-throughput image processing, where millions of objects have to be processed in reasonable time and without user interaction.

### Our contribution

In this contribution, we describe the development and application of a fully automatic image acquisition and processing pipeline. Initialized with a set of 10 intuitively interpretable parameters, the method performs robustly on long-term high-throughput experiments which exhibit a variety of cell shapes (e.g. due to differentiation), numerous cell densities (e.g. due to cell proliferation), and changing image qualities (due to different fields of view and technical alterations during long-term imaging). The strength of this protocol is the combination of changes in image acquisition that are optimal for automatic computation and a set of robust yet fast methods, which allow the processing of hundreds of thousands of images with high accuracy without changing the parameter settings. The protocol has been optimized for parallelized computing of single images. We demonstrate the robustness in cell detection and computational efficiency of our method by processing and analyzing a 6 day high-throughput time-lapse experiment of differentiating hematopoietic stem cells. Using 150 nodes of a computation cluster, we were able to process ∼315,000 images in ∼72 hours, resulting in identification of ∼270,000,000 cells. We evaluate the method on a manually inspected test set of bright field images as well as statistically on the full time lapse experiment. Compared to a pipeline of algorithms available in *CellProfiler*, our method outperforms the standard approach with an overall cell detection accuracy of at least 82%. To demonstrate the predictive power of our approach, we derive population doubling times directly from the computed cell numbers over the whole experiment, which are in accordance to previously reported cycle times for these cell types [20]. In addition, we show that the computed doubling times match up with cell cycle times of 1600 manually tracked cells.

## Results and discussion

### Image acquisition and processing steps

**Figure 1:**
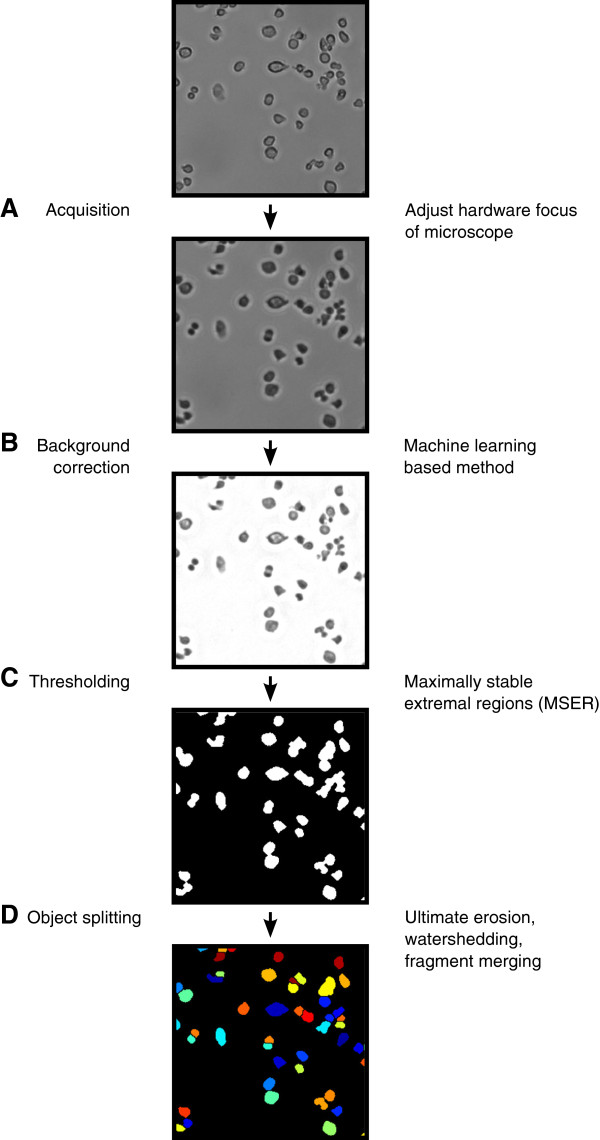
**Flow chart of the proposed method.** Detailed description of our proposed method. The results of each step are exemplified on a bright field image of hematopoietic progenitor cells. **(A)** The image is acquired with the focus set 18*μ*m below the optimal focal plane to enhance contrast of cells. **(B)** The inhomogeneously illuminated background is corrected by a machine learning based approach to resolve differences in illumination across different locations on the cell culture plate and over time. **(C)** Foreground objects are identified by maximally stable extremal region (MSER) detection. **(D)** Splitting of clumped cells. Maxima of cells are identified by ultimate erosion and split by watershedding. Over-segmented cell bodies are reconstructed by merging of too small neighboring regions.

For the development of a method to analyze high-throughput microscopy data, it is especially important to incorporate algorithms that are (i) robust against heterogeneities between images that are processed and (ii) able to process single images in the range of seconds up to a few minutes at maximum in order to finish a full experiment in reasonable time. In this work, we chose the algorithms used in every step according to these requirements. The complete pipeline is visualized in Figure 1.

The first step in our method concerns image acquisition. Adapted from Selinummi et al. [21], we recorded every image with the microscope’s auto focus set 18*μ*m below the optimal focal plane. In the case of bright field image acquisition with a 10x fluar objective (Zeiss), the change of the focal plane resulted in enhanced contrast of single cells, yet with a loss of textural complexity (see Figure 1A). The cell body was evenly illuminated and much darker than the background. In addition, every cell exhibited a bright halo that is supporting the identification of touching cells.

After all images were acquired in the proposed manner, differences in illumination across the images had to be resolved. We used an adapted version of the method proposed by Schwarzfischer et al. [22]. This machine learning based algorithm estimates the background for every image, using a grid of image patches that are classified as showing only background or a mixture of background and foreground pixels. In comparison to standard correction methods like Gaussian filtering that is parametrized on the average foreground object size, the machine learning based method is able to estimate the background more robustly. As shown in Figure 1B, every cell body was clearly separated from the background. The halo surrounding each cell was corrected, yet clumped cells still exhibited a change in illumination at their touching edges. Due to the time-consuming feature calculation during machine learning, this algorithm was occupying nearly 50% of total computing time for a single image, which was in our case 30 to 50 seconds on a standard laptop (Intel Core i7 dual-core, 2.8GHz, 8GB RAM, Windows 7 64bit).

In the next step, all foreground objects had to be separated from the background. In our method, we used the maximally stable extremal regions (MSER) algorithm [23]. An advantage compared to thresholding methods such as Otsu’s method is its robustness in segmentation when there are inhomogeneities in object illumination or huge differences of cell densities between different images. As shown in Figure 1C, MSER correctly identifies nearly all cell bodies. The used implementation of MSER has linear time complexity, thus it is able to process a single image (i.e. 1000x1000 pixels) in milliseconds [24].

Eventually it was necessary to split clusters of multiple cells that were segmented as a single foreground object (i.e. under-segmentation). We used a two-step approach consisting of an initial marker-based watershedding, followed by merging of cells that were erroneously split into fragments (i.e. over-segmentation). In this step (see Figure 1D), the earlier conducted out of focus acquisition was very advantageous: the homogeneous illumination of cell bodies and the slightly brighter interfaces of touching cells simplified the task to find cell centers by ultimate erosion, which then served as seed points for the watershed algorithm. Depending on the number of cells in an image this step occupies 10 to 50 seconds of processing time.

### Application

**Figure 2:**
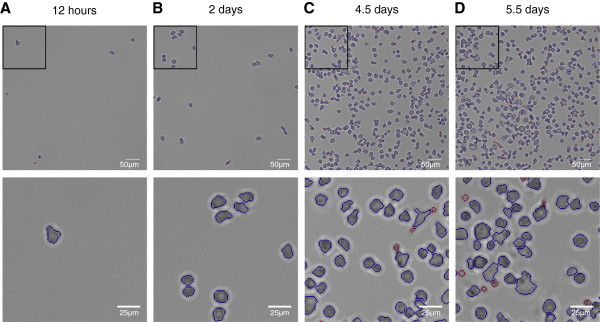
**Manual examination of segmentation results.** Manual examination of segmentation results, shown at exemplary image patches over the whole time span of a 6 day time-lapse experiment of differentiating hematopoietic stem cells. Blue outlines: Segmented objects regarded as cells. Red outlines: Objects unlikely to represent cells (size <50 px and eccentricity >0.99). First row: 500x500px image patch, second row: 150x150px image patch. **(A)** 12 hours after experiment start. Very few cells are populating the field of view. Cell outlines are correctly segmented. Erroneous measurements originate in debris in the image. **(B)** 2 days after experiment start. The number of cells is slightly increased, still the object density is very sparse. Pairs of clumped cells can be identified, which are correctly split by the method. **(C)** 4.5 days after experiment start. More complex cell morphologies arise that lead to errors in segmentation. The field of view becomes more and more crowded, complicating the identification of single cells. Small artifacts that are a result of over-segmented cells or fragments of dead cells are filtered by size. **(D)** 5.5 days after experiment start. Most cells are differentiated and different morphologies can be found. Segmentation errors are observed more frequently, especially for adherent cells with elongated shape.

We applied our method on a time-lapse experiment of hematopoietic stem cells (HSCs) under conditions that promote differentiation towards myeloid cells. For a review of blood cell differentiation see for example Orkin et al. [25]. Two wells of a plastic slide were imaged in intervals of ∼2.3 minutes for 6 days. The wells were sparsely covered with cells at the beginning, yet at the end of the experiment all wells were densely populated by hematopoietic progenitors and differentiated cells (see Figure 2C). To cover the full area of interest in high resolution, each well was divided into a grid of 33 overlapping fields of view (i.e. images covering different areas of a well). Each field of view was recorded once in a time interval. Details regarding experimental conditions and the parametrization of our method to process this experiment are described in the methods section.

Complete processing of the full data set occupied 72 hours, using 150 cores of a computer cluster. The average node architecture was equal to an Intel Xeon 2GHz, 4GB RAM running a 64bit linux-based operating system. Complete processing of a single image with average cell density (see Figure 2) lasted 100 seconds. To account for small debris or fragments of dead cells that were erroneously segmented by our method, we discarded all foreground objects with a size < 50 pixels and an eccentricity > 0.99. The final test set comprised ∼315,000 raw images with the according object masks and computed background corrections, covering ∼270,000,000 identified objects.

### Evaluation

The performance of a segmentation approach can be measured in different ways depending on the analyses that are intended after processing. For the development of automatic tracking approaches it is necessary to identify single cells with high accuracy. Especially cells that stick together shortly after division or clusters of multiple cells need to be split correctly. For population analysis or simple cell counting it suffices to detect the number of cells in each image with high accuracy. Here, we manually determined the total number of cells after 12 hours, 2 days, 4.5 days and 5.5 days at two randomly chosen fields of view per well. We evaluated if a cell was (i) correctly segmented, (ii) missed, (iii) over-segmented or (iv) under-segmented. Next, we computed the average accuracy, specificity and sensitivity of cell detection based on the number of true positives (complete cell bodies, the largest fragment of over-segmented cells and one cell per under-segmented object), false positives (dirt and cell fragments) and false negatives (missed cells, remaining cells in under-segmented objects). In addition, we calculated the mean and the according standard deviation of cell densities (cells per *mm*^2^) over all fields of view at the given time point.

After 12 hours (Figure 2A), all fields of view were sparsely covered with cells (cell density 5.6±2.4 1/*mm*^2^). Despite the high fraction of correctly segmented cells (92%), debris in the examined fields of view that was falsely identified as a cell by our method lead to a decrease in accuracy (83±11*%*). The low number of cells at this early time point resulted in a high variability (11%) between the fields of view.

At day 2 (Figure 2B), the number of cells increased to a density of 16.2±7.1 1/*mm*^2^. Pairs of clumped cells appeared. 82% of all cells were correctly segmented, only very few cells were missed or over-segmented (11%) and under-segmentation was not detected. The cell detection accuracy was 82±3*%*.

At day 4.5 (Figure 2C), the number of cells across the examined fields of view was significantly larger (741.6±250.6 1/*mm*^2^). We observed cells that were clumped together in large clusters and first differentiated cells with more complex morphology were found. Cells with round shapes were correctly identified in most cases (92%), especially round clusters of cells were under-segmented (1%) and cells with elongated shape were over-segmented (4%). Most over-segmented cells were still only counted once since smaller fragments were discarded by the filtering step (see methods). 1% of the cells were missed, mostly because of bad contrast or direct contact to the image border. Due to the large increase in cell number, debris did not significantly contribute to a drop in cell recognition accuracy anymore. The accuracy at this time point was 95±1*%*.

At day 5.5 (Figure 2D), fields of view of both wells were very densely populated by cells (1.2∗10^3^±0.22∗10^3^ 1/*mm*^2^) that were exhibiting a variety of shapes. With 1.2%, the fraction of missed cells was even reduced compared to the time point examined before. Over- and under-segmented cells were observed more frequently (6% and 1%, respectively), yet most cells were correctly segmented (90%). The amount of debris was increased, mostly due to clumps of fragments of dead cells. Yet, the cell detection accuracy was very high (92±3*%*).

Sample images showing the cell densities at different time points for our method are given in Figure 2. The object quantification is summed up in Table 1.

**Table 1:** Manual evaluation of segmentation results

**Experiment time**	**12 h**	**2 d**	**4.5 d**	**5.5 d**
Number of manually counted cells	37	90	5837	7414
Correct	34(91.9*%*)	74(82.2*%*)	5356(91.8*%*)	6638(89.5*%*)
Missed	1(2.7*%*)	4(4.4*%*)	78(1.3*%*)	86(1.2*%*)
Over-segmented	2(5.4*%*)	10(11.1*%*)	230(3.9*%*)	411(5.5*%*)
Under-segmented	0(0.0*%*)	0(0.0*%*)	46(0.8*%*)	87(1.2*%*)
Debris	6	7	1	32
True positives	36	84	5632	7136
False positives	6	13	96	329
False negatives	1	6	205	278
Accuracy	0.83±0.11	0.82±0.03	0.95±0.02	0.92±0.03
Specificity	0.86±0.11	0.87±0.06	0.98±0.01	0.96±0.03
Sensitivity	0.97±0.06	0.94±0.04	0.96±0.02	0.96±0.01

Two randomly chosen fields of view per well were quantified for 12 hours, 2 days, 4.5 days and 5.5 days, respectively. In each field of view, the number of true cells was counted. All segmented objects were classified as correct, over-, under-segmented, or debris. Accuracy, Sensitivity and Specificity of cell detection were calculated based on true positives (complete cell bodies, the largest fragment of over-segmented cells and one cell per under-segmented object), false positives (dirt and cell fragments) as well as false negatives (missed cells, cells in under-segmented objects). Note that we deliberately keep differences in the total number of counted cells at different experiment times, since these impact on the standard deviation of accuracy, specificity and sensitivity.

**Figure 3:**
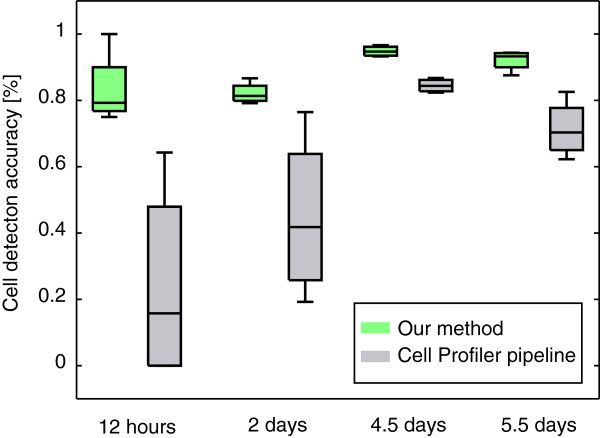
**Comparison of manually evaluated cell detection accuracy.** Comparison of manually evaluated cell detection accuracy as described in Table 1 between our method (green boxplots) and the *CellProfiler* pipeline (gray boxplots). Especially at the two early time points, CellProfiler performs not very robust on the different fields of view. Note that the pipeline was parametrized to perform best on images at day 4.5. Thus, the pipeline might be able to perform well on images on the early time points, but is not robust enough with the given parameter settings.

To demonstrate the superior robustness of our method, we conducted the same manual evaluation with the results from a pipeline of methods available in *CellProfiler*. For details of the pipeline and the parameter settings, see the Methods section. We applied the CellProfiler pipeline on the identical set of out-of-focus images and optimized the parameters of each module based on a single image of day 4.5. For a graphical comparison of the cell detection accuracy of our method against the *CellProfiler* pipeline, see Figure 3.

At 12 hours, the *CellProfiler* pipeline produced highly heterogeneous results. The used thresholding algorithm performed well on images of 2 fields of view but produced completely mis-segmented images on the others, leading to a low cell detection accuracy (24±31*%*). This was most likely due to errors in the clumped cell splitting step.

For images taken at day 2, the *CellProfiler* pipeline performance increased (45±25*%*). Yet the accuracy was rather low and less robust across different fields of view (25%).

At 4.5 days, the increased cell density lead to an improvement in the cell detection accuracy (84±2*%*), with a huge decrease of standard deviation. Still, 3% of the cells were missed completely and 9% were under-segmented.

At the last manually evaluated time point of 5.5 days, cell detection accuracy of the CellProfiler pipeline decreased to 71±9*%*. This was mainly because of the high fraction of missed (20%) and under-segmented (6%) cells.

Taken together, our method showed high robustness in cell detection and low over- and under-segmentation over the full experiment range. Even at very late time points where the wells were very densely covered, the cell detection accuracy was satisfying (∼92%). The out-of-focus acquisition improves the overall segmentation accuracy of our pipeline: Applied to a comparable in-focus movie, the segmentation accuracy dropped to 70% due to over-segmentation of badly contrasted cells and complex cell texture.

As shown in Figure 3, our method clearly outperformed the standard *CellProfiler* pipeline. Note that the low cell detection accuracy in the early time points does not necessarily mean that *CellProfiler* in general is not able to segment this type of images (i.e. very few cells). Still, the combination of algorithms performed less robustly on images with different cell densities, given the parameter set that we optimized for images with medium cell density (i.e. day 4.5).

Finally, we would like to note that our pipeline achieved similar robust results (segmentation accuracy ∼85%) in a second long-term high-throughput experiment.

### Population doubling time derived from cell counts

A possible use-case in the analysis of high-throughput time-lapse experiments is the control of cell proliferation. Due to photo toxicity or different medium conditions, cells could die early or exhibit deviating proliferation rates [5], which would introduce errors in later analyses that are conducted on the data set.

**Figure 4:**
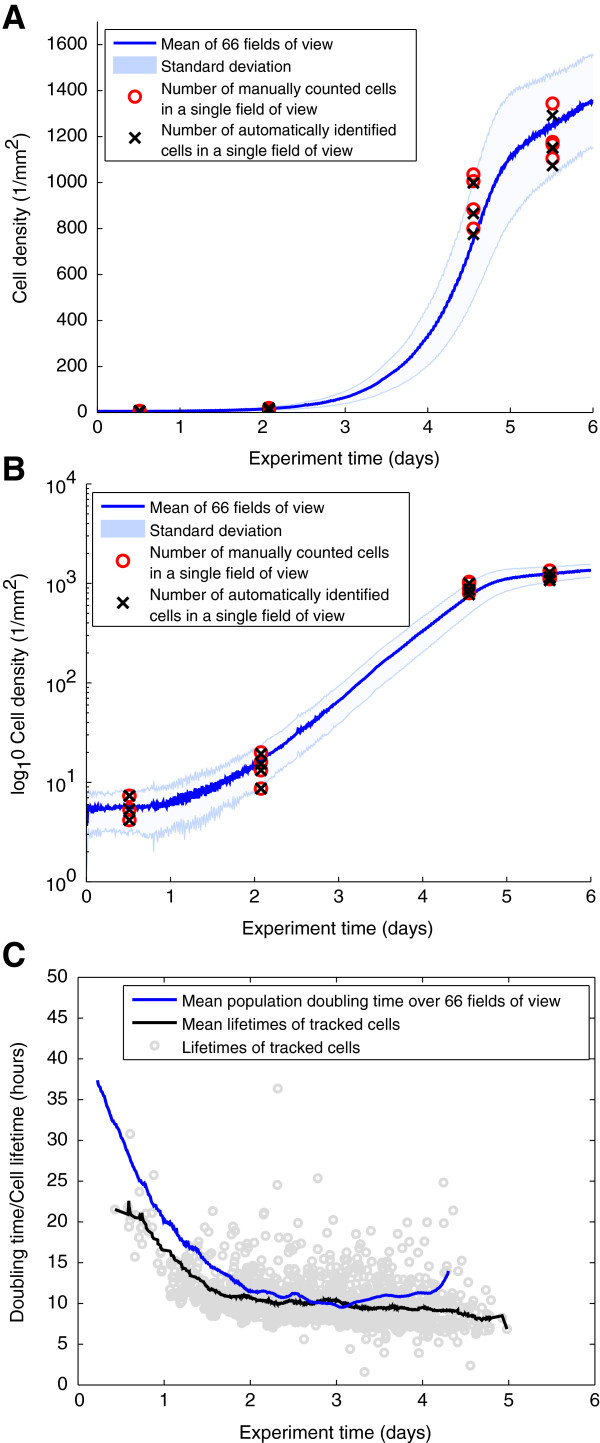
**Whole-movie analysis of population growth rates and doubling times.** Whole-movie analysis of population growth rates and doubling times. **(A)** Mean cell densities over 66 fields of view (blue line) and according standard deviation (light blue patch) per *mm*^2^ covering the full experiment time range. Red circles indicate the manually determined number of cells in 4 randomly chosen fields of view at 12 hours, 2 days 4.5 days and 5.5 days as described in Table 1. **(B)** Increase of cells plotted in log-scale. A non-exponential growth phase could be identified until day 2. Between day 2 and day 5 the number of cells increased exponentially. **(C)** Population doubling times (blue line) per time point and cell cycle times of ∼1600 manually tracked cells with mean lifetimes from 0.5 to 5 days (gray circles). The doubling time decreased until day 2, where it roughly stabilized around 9 to 11 hours. The cell cycle times coincided with the derived doubling times, indicating a correct automatic derivation.

Here, we first analyzed the mean cell density over 66 fields of view over the full experiment time span (blue line in Figure 4A). We found that the number of cells increased monotonously until a plateau roughly at day 5. We compared the results with the manually quantified numbers of cells as shown in Table 1 and found them to reside within the standard deviation of the number of objects. From our accuracy estimation in Table 1, we conclude that the plateau is not due to a failure of our method, but resulted from biological or experimental reasons. One explanation could be the differentiation and thus post-mitotic state of the hematopoietic cells, but also a depletion of the medium. In addition, the high density of cells could lead to an arrest in population growth.

Plotting the growth curves in log scale (see Figure 4B) revealed three different phases of population dynamics. At the beginning of the experiment, the number of cells increased sub-exponentially. Between approximately 2 and 4.5 days a clearly exponential increase with an average doubling time of 10-12 hours was observed. The population stops to grow exponentially and reaches a plateau after ∼4.5 days.

Based on the cell counts resulting from our image processing method, we derived the population doubling time. Due to the high temporal resolution of ∼2.3 minutes between measurements, the population doubling time could be estimated by computing the difference of each time point and the time point where the cell number had doubled, respectively (see blue line in Figure 4C). The doubling time decreased from ∼40 hours and stabilized after day 2 until day 4 at around 10 to 12 hours.

To validate the estimated doubling times, we tracked 1600 cells manually using our in-house developed software TTT [7]. As shown in Figure 4C, the cell cycle times of tracked cells that were born between 0 and 4.5 days (gray circles) show the same trend, decreasing from ∼20 hours to 9-11 hours in the exponential growth phase.

## Conclusions

In this contribution, we described a fully automated method for processing of high-throughput bright field microscopy experiments, that relies on the combination of optimized image acquisition and a concatenation of image processing algorithms that identify cells in in a robust yet time efficient manner. Using the same parameter set for all images, we applied the method on a 6 day time-lapse movie of differentiating hematopoietic stem cells and achieved a cell detection accuracy of at least 82%, which outperformed a pipeline of algorithms available in *CellProfiler*. We demonstrated the application of the results generated by our method by computing population doubling times based on the increasing number of cells over the whole experiment in time and space. We compared the results to the cell cycle times of 1600 manually tracked cells and showed that the automatically derived doubling times coincided with the manually tracked cell cycle times.

The full data set of ∼315,000 images was processed within ∼72 hours. Note that this value was achieved by parallel computing on 150 cores of a computation grid. However, the code used in this work was not optimized for speed. Using implementations in C++ or Java that are optimized for fast computation, the processing time could be further improved. This would allow on-line processing of a time-lapse or high-content experiment during acquisition, which offers powerful options. For example, a researcher could check population doubling times, and thus cell health during a time-lapse experiment, or acquisition could be stopped automatically when a certain number of cells is reached in the experiment.

The robustness of our method relies on the out of focus acquisition of bright field images, which results in very well defined cell outlines but also in the loss of textural complexity for single cells. However, by acquiring an additional image with an optimally set focus in every interval, the quantification of morphological features such as shape and texture becomes feasible. Together with the high accuracy in cell detection of our method, this will support the development of automatic tracking approaches in time-lapse microscopy. For both time-lapse and high-content screening experiments, morphological quantification of millions of cells in one experiment allows the application of machine learning methods to classify, e.g., dying and surviving cells after drug treatment or the fate of differentiating stem cells.

Our pipeline will be improved and adapted in the future. A promising avenue is the extension of the MSER segmentation algorithm to include more cellular features, like eccentricity or size.

Another possible improvement in our pipeline is the splitting of clumped cells. Many methods have been developed in the past, e.g. ellipse fitting that is well suited to split nuclei or cells with round morphology [26]. Unfortunately, the restrictive assumptions in this method do not allow more complex cell shapes that may emerge during a long-term movie of differentiating cells. We showed that our method performs well at the segmentation of hematopoietic stem and progenitor cells, which show round morphology. Still, the marker based watershedding we used is flexible enough to also cover more complex cell shapes that are appearing later in the differentiation process. Li et al. proposed a method based on gradient flow tracking and showed that it performs well on fluorescent images with hundreds of stained nuclei that are densely packed and are thus exhibiting different morphologies [27]. Another approach could include the development of a robust and fast performing level set evolution method. This class of algorithms has already been shown to perform very well on complex cell shapes [17], however the computional complexity hinders an application in a high-throughput context. An approach that was already applied on high-throughput screens is to iteratively learn the different cell shapes of a given cell type or system in an experiment [28]. Due to the modular structure of our method, the extension with algorithms that are able to split cells with a more complex morphology is easily possible. In the time-lapse experiment that was used in this work, these improvements could specifically enhance the cell detection accuracy for differentiated blood cells at the end of the experiment.

In summary, we believe that the high overall robustness in cell detection as well as the fast processing speed of our method will be of great service for the analysis of high-throughput microscopy experiments.

## Methods

### Experimental setup

Femurs, tibiae and ilia of a healthy mouse strain on C57Bl/6 background with no discernible phenotype were removed from 14 weeks old mice and bone marrow was extracted. Cells were isolated by fluorescence activated cell sorting. According to the original publications, the fraction of true HSCs that are purified by this method is between 40%-60% [29,30]. Directly after sorting, the cells were plated out on a plastic slide (*μ*-slide VI coated with Fibronectin, Integrated BioDiagnostics GmbH, Munich, Germany) with two physically separated wells in serum-free medium (StemSpan SFEM, StemCell Technologies) supplied with cytokines that only promote differentiation towards myeloid cells. All animal experiments were performed in compliance with the institutional guidelines of the Helmholtz Center Munich and the regulations of the State of Bavaria.

### Image acquisition

Due to the cameras limited field of view, both wells were subdivided into 33 overlapping tiles (fields of view). Each field of view corresponds to an image of 1388x1040 pixels that was saved in 8-bit png format. Imaging was conducted using a Cell Observer microscope (Zeiss) surrounded by an incubator to maintain a constant temperature of 37°C. Images were obtained using a 0.63x TV-adapter (Zeiss) and an AxioCam HRm camera (Zeiss), with a 10x fluar objective (Zeiss). Each field of view was imaged in intervals of ∼2.3 minutes for 6 days. Automatic focusing was achieved using a hardware autofocus (Zeiss) which was set to 18 *μm* below the optimal focal plane to acquire slightly blurred images. The complete data set comprised a total of 315,942 images (4787 time points * 66 fields of view) and occupied ∼500 gigabytes of hard drive space.

### Image processing

To resolve differences in illumination across different fields of view and over time, all images were background corrected using an adapted version of the machine-learning based method developed by Schwarzfischer et al. [22]. The method subdivides a given image *I*(*x*,*t*) with space coordinates *x*=(*x*_1_,*x*_2_) at time point *t* into overlapping tiles of equal dimensions (here: 30 x 30 pixels). For each tile the statistical moments of the intensity distribution are calculated. The tiles are then split into two groups by density-based clustering [31,32]. The mean intensity of tiles classified as belonging to background are used as seed-points to inter- and extrapolate the full background image *B*(*x*,*t*). The corrected image was derived by dividing the raw image *I*(*x*,*t*) by *B*(*x*,*t*). Halos were corrected by setting all pixels that were brighter than the background to the background intensity.

Foreground objects were identified by Maximally Stable Extremal Regions (MSER) [23,24]. The algorithm is a feature detector, originally designed to find informative regions in two images of the same scene which were taken under different conditions or arbitrary viewpoints. MSER achieves segmentation by thresholding, i.e. given a threshold *τ*, the method classifies each pixel in an image to belong to the foreground or background. By iterating over every possible discrete value for *τ*, a set of connected components is generated. Given that *τ*=0 would result in all pixels being regarded as background, the foreground regions will grow bigger the higher *τ* is set. The components that do not change over an interval *Δ* of different values for *τ* are regarded as stable extremal regions. An implementation with linear complexity is used that requires 4 parameters: *minSize*, the minimal size in pixels of a region to be regarded as foreground; *maxSize*, the maximal size in pixels of a region to be regarded as foreground; *maxVariation*, the maximal Variation inside a region according to the value of *Δ*. If the algorithm reported nested regions in an image, these regions were merged, resulting in a binary mask *BW*(*x*,*t*). Holes in foreground regions, i.e. background pixels with no connection to an image border, were filled. Note that in the used formulation, the MSER algorithm is only applicable for 8-bit images.

In a last processing step, foreground objects that were likely to represent mitotic cells or cell clumps were separated using a modified version of of marker based watershedding.

First, the raw input image *I*(*x*,*t*) was inverted resulting in local intensity maxima residing at cell centers. All local maxima were extracted to a binary mask *BW*_*max*_(*x*,*t*), which was done calculating the regional maxima of the Euclidean distance transform of the cell mask *BW*(*x*,*t*) (i.e. ultimate erosion as implemented in the MATLAB image processing toolbox). In a next step, the distance transform *I*_*dist*_(*x*,*t*) of the foreground mask *BW*(*x*,*t*) was computed. The final transformed image *I*_*transformed*_ was derived by imposing *BW*_*max*_ on *I*_*dist*_. Watershedding [33] was applied on *I*_*transformed*_, resulting in the mask of identified and split objects *BW*_*watershed*_.

To reduce over-segmentation, a rule-based split & merge procedure of small regions from *CellProfiler* 1.0 was applied [11]. A list of adjacent neighbors for all objects in *BW*_*watershed*_ that are likely to be over-segmented (i.e. very small objects with high eccentricity) was computed. For each neighbor the following criteria were evaluated: (i) For the pixels residing on the interface of the evaluated object and its neighbor, the likelihood to belong to the background or to the foreground was computed. Foreground and background were represented as Gaussian distributions, where mean and variance are derived from the image (i.e. pixels that were classified as foreground and background by the thresholding step). (ii) The eccentricity for the merged object was calculated. The evaluated object and its neighbor were merged if the interface pixels were more likely to belong to the foreground and if the merged object’s eccentricity was lower than an empirically determined value (here: 0.7).

For each identified object in *BW*_*split*_, its area in pixels and eccentricity were quantified. To clean the data set from objects that were unlikely to represent cells, foreground regions with size <50 pixels or eccentricity >0.99 were discarded. Both values were empirically determined based on randomly drawn images of the data set. In addition, all objects that were touching an image border were removed. The final mask of identified cells *BW*_*cells*_ is the output of this method.

### Parameter settings

For the dataset that was used in this work, the method was initialized using the following parameter set (one setting for all images of the data set): background correction: *tiledimensions* = 30x30 px, *overlap* = 15 px, *eps* = 0.1, *MinPts* = 6; MSER thresholding: *λ* = 5, *minSize* = 30 px, *maxSize* = 4000 px, *maxVariation* = 1; clumped cell splitting: *MaxEccentricity* = 0.7, *minSize* = 30 px, *maxSize* = 1000. The data set was split into junks of 150*2489 images. All junks were processed in parallel on a node of a computation cluster.

### CellProfiler pipeline

We used *CellProfiler* version 2.0 (r11710) [11] and created a pipeline for automatic processing of the bright field images that we used in the manual evaluation. The following modules were sequentially called for each image: Correct Illumination (Gaussian filter, Average object size: 60 px), Apply threshold (Otsu global), Identify primary objects (Typical diameter of cells: 5 to 50, splitting method: Intensity, method to draw dividing lines: Shape), Convert objects to image (saved binary mask). Parameters were optimized according to a single image of the evaluation set of day 4.5. In the case of long-term time-lapse experiments the constant change of cell density and illumination, as well as the acquisition of different fields of view makes Otsu’s method the best choice out of the algorithms that are available in CellProfiler.

### Evaluation

Manual quantification of segmentation results was done using the java based image processing tool ImageJ 1.47K with the plugin CellCounter [34].

### Implementation and parallelization

All methods were implemented using MATLAB version 8.0.0.783 (R2012b) with the additional packages image processing toolbox 8.1 and statistics toolbox 8.1. If MATLAB code was available for the cited methods, this code was used. For MSER thresholding, a C++ implementation with linear time complexity was used. To speed up computation times, the data set was split into junks of images and processed on a computation cluster (sun grid engine version 6.2u5). The average node architecture was equal to an Intel Xeon 2GHz, 4GB RAM running a 64bit linux-based operating system.

## Availability

The code of our pipeline is available as Additional file 1.

## Competing interests

The authors declare that they have no competing interests.

## Authors’ contributions

FB developed the method and conducted the analyses. FB and CM wrote the manuscript. PH conducted the time-lapse experiment and manual tracking. MS contributed the implementation for background correction. OH contributed the MSER implementation. CM, TS and FT designed and supervised the study. All authors read and approved the final manuscript.

## Supplementary Material

Additional file 1 **Matlab *code *of the presented method.** Click here for file
