# Supplementary figures and images for: An automatic method for robust and fast cell detection in bright field images from high-throughput microscopy
Source: BMC Bioinformatics. 2013 Oct 4;14:297. doi: 10.1186/1471-2105-14-297 (PMC3850979; doi:10.1186/1471-2105-14-297)

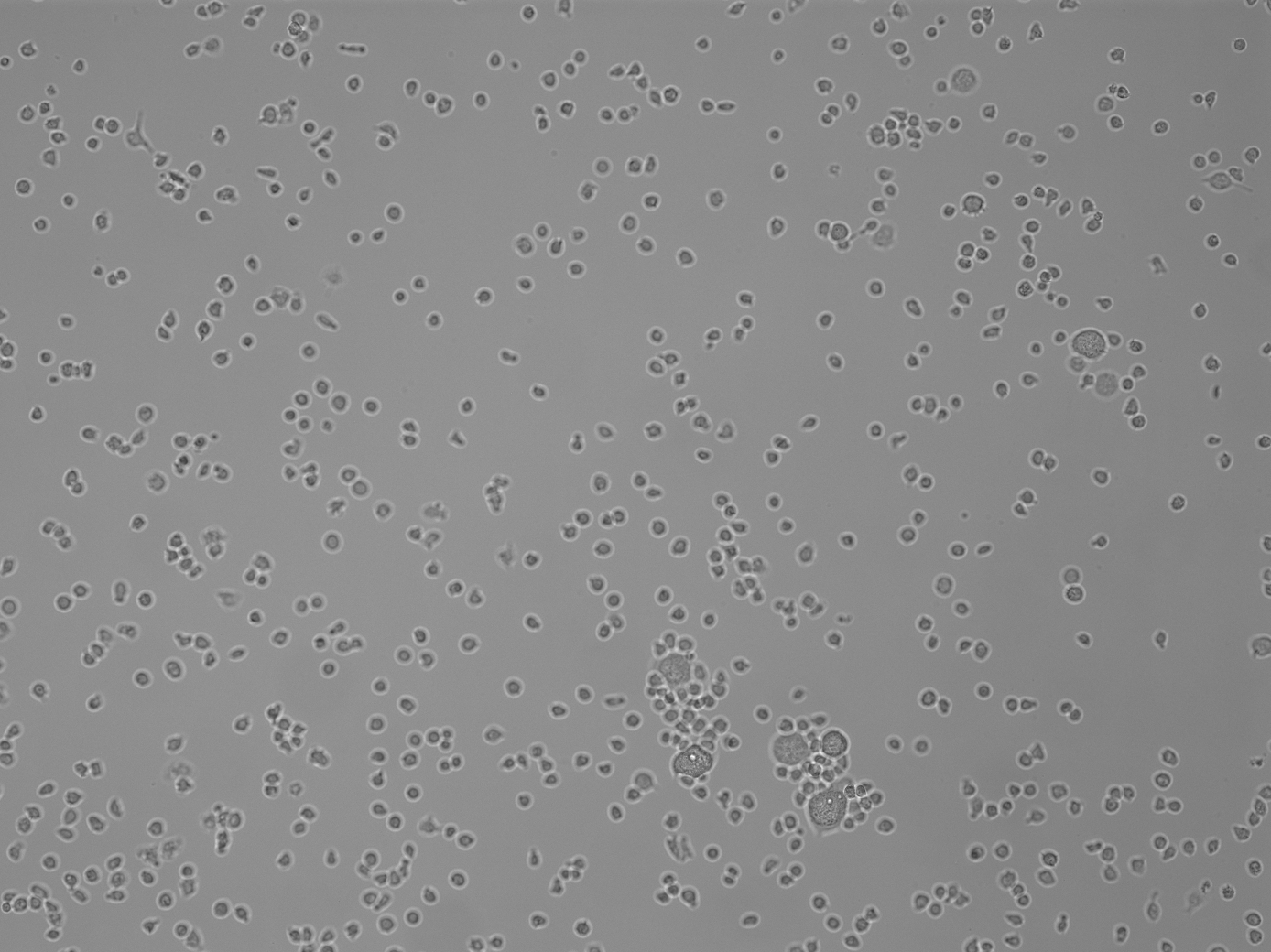

Supplement: Additional file 1 — Matlab code of the presented method. [file 1471-2105-14-297-S1.ZIP › Demo1.png]
